# Inactivation of multidrug-resistant bacteria using cold atmospheric-pressure plasma technology

**DOI:** 10.3389/fmed.2025.1522186

**Published:** 2025-03-05

**Authors:** Xingxing Wang, Mengzhen Chen, Ye Lu, Peihao Yu, Chen Zhang, Chao Huang, Zhibiao Yang, Yan Chen, Jian-cang Zhou

**Affiliations:** ^1^Department of Critical Care Medicine, Sir Run Run Shaw Hospital, Zhejiang University School of Medicine, Hangzhou, Zhejiang, China; ^2^Key Laboratory of Microbial Technology and Bioinformatics of Zhejiang Province, Hangzhou, China; ^3^Department of Infectious Diseases, Sir Run Run Shaw Hospital, Zhejiang University School of Medicine, Hangzhou, China; ^4^Regional Medical Center for National Institute of Respiratory Diseases, Sir Run Run Shaw Hospital, Zhejiang University School of Medicine, Hangzhou, China; ^5^Panasonic Home Appliances (China) Co., Ltd., Hangzhou, China

**Keywords:** multidrug-resistant bacteria, cold atmospheric plasma, sterilization, electron microscopy, nosocomial infection

## Abstract

**Objective:**

This study aimed to investigate the impact of cold atmospheric-pressure plasma (CAP) on multidrug-resistant (MDR) bacteria on various surfaces under nosocomial circumstances and the underlying mechanism.

**Method:**

Four common MDR bacteria (carbapenem-resistant *Acinetobacter baumannii*, carbapenem-resistant *Pseudomonas aeruginosa*, methicillin-resistant *Staphylococcus aureus*, and carbapenem-resistant *Klebsiella pneumoniae*) were inoculated on nosocomial surfaces, which were subsequently exposed to CAP. Then the bacteria from surfaces were recovered and diluted. The killing curve was analyzed to evaluate the sterilization effects of CAP. Electron microscopy was used to evaluate the changes in cell morphology.

**Result:**

In the CAP-producing device, most of the MDR bacteria were nearly inactivated after 2 h of CAP treatment. Under the simulated ward, CAP exerted an inactivating effect on MDR bacteria. Scanning electron microscopy revealed that the surface of MDR bacteria became blurred, the bodies ruptured and adhered to each other after CAP treatment. The cell walls were thinner as revealed by transmission electron microscopy.

**Conclusion:**

CAP could inactivate the most common MDR bacteria on nosocomial surfaces in simulation ward settings by destroying the structure of pathogens. Our data provided insights into the sterilization of MDR bacteria using CAP and suggested a novel in-hospital disinfection alternative.

## 1 Introduction

The incidence of hospital-acquired infections (HAIs) has grown exponentially on a global scale since 1980 and has become a challenge for clinicians owing to the emergence and dissemination of nosocomial multidrug-resistant (MDR) bacteria (1). Controlling the escalation of MDR HAIs has emerged as a paramount concern for global public health. There is an imminent demand for the development of alternative strategies to complement antibiotic therapies to combat clinical MDR bacterial infections. Common routes of MDR bacteria transmission in hospitals include patients colonized by MDR bacteria staying for extended periods and disseminating them to neighboring patients, contaminated clinical equipment that cannot be disinfected owing to heat sensitivity, and inadequate disinfection procedures by hospital staff (2).

One of the cornerstone strategies in traditional disinfection involves the use of disinfectants, such as chlorhexidine, silver salts, glutaraldehyde, peroxides, and ortho-phthalaldehyde (3). However, conventional approaches have several limitations. Notably, disinfectants require a minimum exposure duration of 5–10 min, with some capable of inducing skin, ocular, and respiratory irritation (4). Hence, numerous innovative alternative strategies have emerged, including hydrogen peroxide vapor, exposure to ultraviolet (UV) light, self-disinfecting surfaces, and cold atmospheric-pressure plasma (CAP) (5). However, UV disinfection and aerosol methods are restricted to vacant rooms and are impractical for daily cleaning routines, thereby unequivocally extending the essential time for bed turnover in healthcare facilities (6). With regard to self-disinfecting surfaces, it is imperative to evaluate the sustained long-term effectiveness of their antimicrobial properties while addressing the formidable challenge of their substantial implementation costs on a broad scale (6, 7).

Considering these factors, CAP has garnered increasing attention as an efficacious disinfection solution with applications across diverse domains, including the medical industry (8) and food production (9). During the COVID-19 pandemic, CAP was confirmed as an affordable, environmentally friendly, and sustainable mask disinfection technology (10). Georg Daeschlein et al. studied the efficacy of argon-based CAP in inactivating 24 strains of pathogens in the skin or wounds of 11 patients, with 11 of 17 MDR pathogens (64.7%) and 5 of 7 other pathogens (71.4%) being completely eradicated (11). In addition, a research study by Thomas Maho et al. found that CAP acted at close range on bacteria and successfully inactivated not only single MDR bacteria, but also showed effect of sterilization on samples inoculated with a mixture of three MDR bacteria as well as on mature colonies (12). CAP is an ideal and versatile instrument with extensive application potential that facilitates the management of MDR HAIs. However, owing to the intricate composition of the active elements in CAP, the precise mechanism underlying bacterial inactivation remains unclear. A previous study demonstrated that CAP inactivates Gram-negative and Gram-positive bacteria via distinct mechanisms (13). Furthermore, certain studies have posited that the involvement of reactive oxygen species (ROS) generated by CAP is pivotal in the process of bacterial inactivation (14). CAP also produces UV light and transient electric fields (EF), which may also play a role in inactivating bacteria (12). In particular, transient EF may lead to changes in bacterial activity by affecting cell membrane permeability. A study demonstrated that CAP can serve as a physical drug delivery vehicle for human cervical cancer HeLa cells and murine breast carcinoma 4 T1 cells by acting on the cell membrane (15). In addition, Thai-Hoa Chung et al. first applied CAP-treated phosphate buffer in combination with microsecond pulsed EF to explore the effects on cancer cells, and showed that this approach resulted in a significant increase in cell membrane electrical permeability even at very low EF strengths (16). E. Robert et al. were the first to employ a nonintrusive and nonperturbative time resolved bi-directional EF measurement method to analyze the propagation of EF generated by CAP within long dielectric tubes (17). To date, the definitive efficacy of CAP in eradicating clinically prevalent MDR bacteria, including carbapenem-resistant *Acinetobacter baumannii* (CRAB), carbapenem-resistant *Pseudomonas aeruginosa* (CRPA), methicillin-resistant *Staphylococcus aureus* (MRSA), and carbapenem-resistant *Klebsiella pneumoniae* (CRKP), has not been conclusively established (18).

In this study, we assessed the efficacy of CAP against MDR bacteria in a hospital setting. Additionally, we investigated the mechanism underlying the CAP-mediated inactivation of MDR bacteria using electron microscopy. These findings enabled the establishment of a scientific foundation for the use of CAP in sterilization procedures.

## 2 Methods

### 2.1 Bacterial strains and culture conditions

CRAB, CRPA, CRKP, and MRSA were all derived from clinical isolates preserved in our laboratory (Table 1). The bacterial cultures were cultivated in trypsin soy broth with shaking at 37°C.

**Table 1:** Bacterial species used in this study and their properties.

Bacterial species	Sequence type (ST)	Resistance phenotype	Resistance genes
*Klebsiella pneumoniae* (CRKP)	ST11	ETP, IPM, MEM, CAZ, ATM, SCF, CIP	*aadA2*, *bla*_CTX-M-65_, *bla*_KPC-2_, *bla*_SHV-158_, *bla*_TEM-1_, *catA2*, *fosA6*, *oqxA*, *oqxB*, *rmtB1*
*Acinetobacter baumannii* (CRAB) (34)	ST90	IPM, MEM, FEP, CAZ, PRL, PTZ, SAM, SCF, ATM, AK, CN, CIP, MH, SMX/TMP, C	*bla*_adc-30_, *bla*_oxa-23_, *bla*_oxa-66_, *aac(2′)-Ib*, *aph(6)-Id*, *aph(3″)-Ib*, *aac(6)-Ib*, *aadA1*, *aacC1*, *aphA1-IAB*, *armA*, *tetA*, *catB6*, *sul1*, *adeABC*, *adeIJK*, *abeM*, *adeT*
*Pseudomonas aeruginosa* (CRPA) (35)	ST463	PIP, FEP, CAZ, PTZ, CZA, IPM, MEM, AZT, AZA	*bla*_AFM-2_, *bla*_KPC-2_
*Staphylococcus aureus* (MRSA)	ST8	OXA, FOX, LEV, CIP	*mecA*

CRKP, carbapenem-resistant *K. pneumoniae*; CRAB, carbapenem-resistant *A. baumanii*; CRPA, carbapenem-resistant *P. aeruginosa*; MRSA, methicillin-resistant *S. aureus*; ETP, ertapenem; IPM, imipenem; MEM, meropenem; CAZ, ceftazidime; ATM, aztreonam; SCF, cefoperazone-sulbactam; CIP, ciprofloxacin; FEP, cefepime; PRL, piperacillin; PTZ, piperacillin/tazobactam; SAM, ampicillin-sulbactam; AK, amikacin; CN, gentamicin; MH, minocycline; SMX/TMP, sulfamethoxazole-trimethoprim; C, chloramphenicol; PIP, piperacillin; CZA, ceftazidime/avibactam; AZT, aztreonam; AZA, aztreonam/avibactam; OXA, oxacillin; FOX, cefoxitin; LEV, levofloxacin.

### 2.2 Structure of the CAP device

The CAP device used in this study was the charged water particle-generating device (Panasonic). The device consists of three components (Supplementary Figure 3): the rod-shaped atomizing electrode, Peltier element, and opposite electrode. In order to generate CAP, the Peltier element of the charged water particle-generating device cools the atomizing electrode so that the water molecules in the air condense the atomized condensate and make it converge to the tip of the rod-shaped atomizing electrode, and then a high voltage (approx. 4,000 V) is applied to the atomized condensate so that the atomized condensate is continuously split under the action of the high-voltage discharge electric field until it generates nano-sized water particles (5 to 20 nm) containing a large amount of highly reactive hydroxyl radical (•OH; Supplementary Figure 4).

The advantage of this device is that this element produces a larger number of nanometer water ions (orders of magnitude up to trillions per second). Due to the encapsulation of water particles, the internal highly reactive •OH exists for more than six times longer than ordinary negative ions, which extends the purge time.

During the experiments, the samples were placed in an environment fitted with a charged water particle-generating device. The input voltage of the power supply was maintained at 5 V, and the treatment time ranged from 0 to 3 h. In the simulation-ward experiment, two wards of 75 m^3^ were selected. The doors and windows were closed, and the CAP generator was installed at the air conditioning outlet. Additionally, we controlled the indoor temperature at 25°C using air conditioning. The humidity in the ward was set to 50 ± 10%. In order to avoid UV from natural light outside the window from entering the room to inactivate the bacteria and interfering with the results of the study, we closed the doors and windows and shut the curtains. According to the computer-simulated heat map of CAP concentration distribution, one point was selected in the high concentration area and another point in the low concentration area for the experiment. The samples of the four MDR bacteria were placed at the high concentration point near the CAP generator (point A) and at the low concentration point far away from the generator (point B). Point A was positioned 1.5 meters away from the CAP device with a height set at 1.3 meters. Point B was located at the entrance of the ward with a height also set at 1.3 meters (Supplementary Figure 5). To ensure that the concentration of CAP reached a steady state, the CAP generator and air conditioner were operated for 24 h at the beginning of the ward experiments before the samples were placed in the room for the experiments, and the samples were taken after 0 h, 2 h, 8 h, and 24 h of treatment, respectively.

### 2.3 Inoculation and treatment of culture dish and fabric surfaces

The overnight bacterial suspension was adjusted to an optical density (OD_600_) of 0.5 (approx. 10^8^ CFU/mL) with sterile phosphate-buffered saline (PBS). The cell density of the adjusted bacterial suspension was then diluted to approximately 10^7^ CFU/mL. Then, 0.01 mL of bacterial suspension was inoculated on the surface of a sterile culture dish (*φ* = 90 cm) or sterilized fabric (2 cm × 2 cm). The initial bacterial concentration on the surface of a culture dish or fabric was approximately 10^6^ CFU/mL. Cells were then treated with CAP for the corresponding times. In addition, we set up a control group by placing only culture dish or fabric with bacterial suspension in the room next to the experimental group, turning on the air conditioning but not placing CAP generators.

### 2.4 Bacterial recovery and enumeration

After the CAP treatment, the bacterial solutions from different material surfaces were recovered in 10 mL PBS, shaken, and mixed well. The recovery solution was diluted with a concentration gradient of 10^0^–10^−7^, then 10 μL of the bacterial solution was taken from each gradient and dropped on agar plates. The agar plates were cultured at 37°C overnight, and clones were counted. The following formula (Equation 1) was used to calculate the concentrations, as follows:


(1) $$\frac{CFU}{\mathrm{mL}}=CFU\times 100\times \mathrm{dilution multiple}\times 10\;\mathrm{mL}\;PBS$$

Bacterial counts were expressed as log CFU/mL.

### 2.5 Scanning electron microscopy

SEM was used to observe cell surface morphology. Bacterial samples treated with CAP for 3 h were subjected to SEM analysis, and untreated bacteria served as controls. Bacterial cells were collected via centrifugation at 6,000 rpm for 3 min and washed 3 times with PBS. The pelleted cells were fixed with 2.5% glutaraldehyde solution overnight at 4°C and then washed 3 times with PBS (15 min each). Next, the samples were fixed with a 1% osmium solution for 1.5 h and washed 3 times with PBS. The samples were dehydrated with a gradient concentration of ethanol solution (50, 70, 90, and 100%) for 15 min each and dehydrated again with 100% ethanol for 20 min. The samples were then dried to the critical point. The samples were observed using a Nova Nano 450 SEM (Thermo FEI, Czech Republic).

### 2.6 Transmission electron microscopy

TEM was performed to detect the effects of CAP treatment on the internal structure of the cells. Samples treated with CAP for 3 h were subjected to TEM analysis and collected through centrifugation at 6,000 rpm for 3 min. The collected cells were washed 3 times with PBS and fixed with a 2.5% glutaraldehyde solution. The next day, the fixed cells were washed twice with PBS and fixed with a 1% osmium solution for 1.5 h. Next, the samples were rinsed 3 times with water (10 min each) and stained with 2% uranyl acetate for 30 min. The cells were then dehydrated using a gradient concentration of ethanol solution (50, 70, 90, and 100%) and washed twice with acetone (20 min each). Dehydrated samples were immersed in a resin overnight at 25°C and then incubated at 65°C for 48 h for polymerize. The samples were sliced using an EM UC7 ultramicrotome (Leica, Germany). Finally, the stained samples were examined using a Tecnai G2 spirit 100 kV TEM (Thermo FEI, Czech Republic). Bacteria without CAP treatment served as negative controls.

### 2.7 Statistical analysis

Data analysis was performed using the GraphPad Prism 9 software. An unpaired two-tailed Student’s t-test was used to compare samples between two groups. Treatment values are presented as means ± standard deviation, and *p* < 0.05 was considered statistically significant.

## 3 Results

### 3.1 CAP inactivation of MDR bacteria on culture dish surfaces

**Figure 1:**
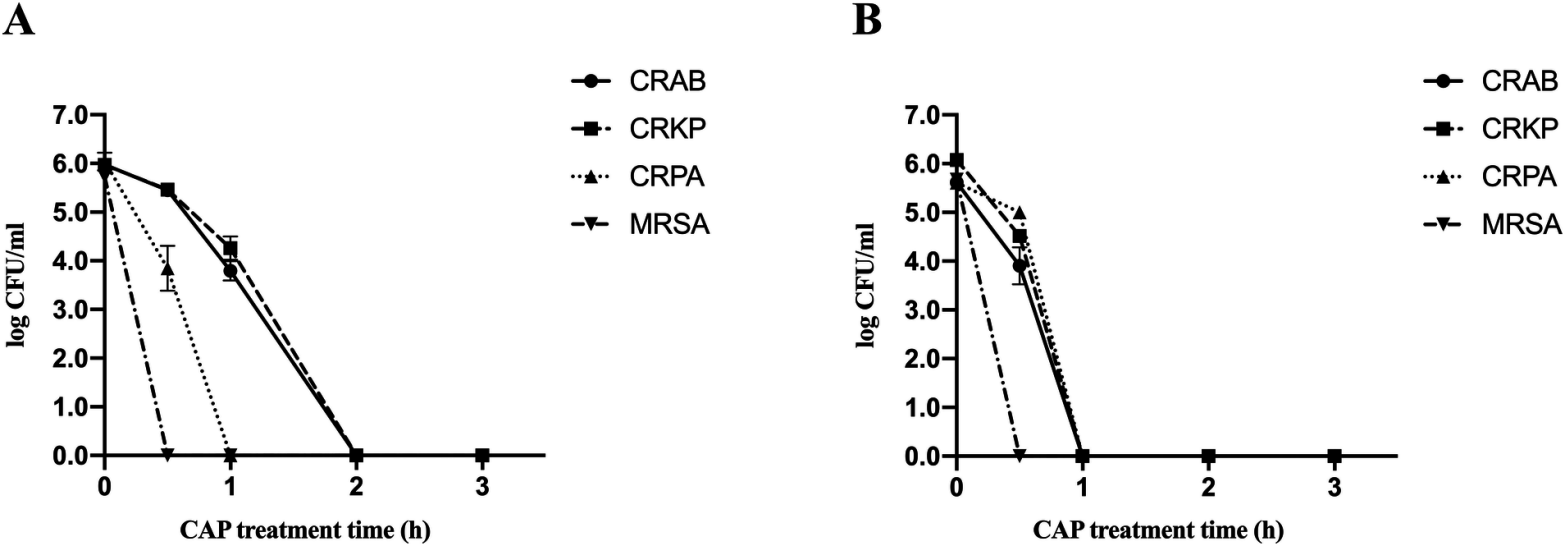
Survival curves of four MDR bacterial species present on culture dish surfaces **(A)** and on fabric surfaces **(B)** after exposure to CAP identified by standard colony counting. Error bars represent the standard error of the mean. CRKP, carbapenem-resistant *Klebsiella pneumoniae*; CRAB, carbapenem-resistant *Acinetobacter baumanii*; CRPA, carbapenem-resistant *Pseudomonas aeruginosa*; MRSA, methicillin-resistant *Staphylococcus aureus*.

To investigate the effect of CAP on the inactivation of MDR bacteria on culture dish surfaces, samples from the experimental group were exposed to the CAP environment and their corresponding control groups were placed in a room without CAP for observation and subsequent calculation of survival rates. Following 30 min of CAP treatment, the survival rates of all MDR bacteria decreased substantially compared to those of the control strains (Figure 1A; Table 2). The most pronounced inactivation was observed in the MRSA group (5.73 ± 0.00 log steps), whereas the least reduction was noted for CRKP (0.50 ± 0.13 log steps). Similar inactivation of other MDR bacteria, including CRAB (0.52 ± 0.08 log steps) and CRPA (2.20 ± 0.46 log steps) was achieved. Upon doubling the treatment duration to 1 h, the inactivation rates of CRAB, CRPA, and CRKP exhibited notable increases. The most substantial inactivation was observed for CRPA (6.04 ± 0.00 log steps), while the most modest reduction was recorded for CRKP (1.71 ± 0.24 log steps). When the treatment time was extended to 2 h, all MDR bacteria were completely absent. These results show that CAP exhibits bactericidal activity against MDR bacteria on culture dish surfaces.

**Table 2:** Reduction rates of all MDR bacterial species present on different surfaces included in the study with CAP treatment.

Bacterial species	Reduction (log CFU/ml; mean ± S.D.)	
	Culture dish surfaces	Fabric surfaces
After 0.5 h of CAP treatment		
CRAB*	0.52 ± 0.08^i^	1.71 ± 0.38^iii^
CRKP*	0.50 ± 0.13^i^	1.56 ± 0.04^iii^
CRPA*	2.20 ± 0.46^i^	0.61 ± 0.13^iii^
MRSA	5.73 ± 0.00^i^	5.67 ± 0.00^iii^
After 1 h of CAP treatment		
CRAB*	2.18 ± 0.20^ii^	5.62 ± 0.00^iv^
CRKP*	1.71 ± 0.24^ii^	6.08 ± 0.00^iv^
CRPA	6.04 ± 0.00^ii^	5.61 ± 0.00^iv^
MRSA	5.73 ± 0.00	5.67 ± 0.00

S.D., standard deviation; CRKP, carbapenem-resistant *K. pneumoniae*; CRAB, carbapenem-resistant *A.baumanii*; CRPA, carbapenem-resistant *P. aeruginosa*; MRSA, methicillin-resistant *S. aureus*. Significant differences (*p* < 0.05) are defined as: ^i^the number of bacteria on culture dish surfaces with significant reduction after 0.5 h CAP treatment compared with control samples; ^ii^the number of bacteria on culture dish surfaces with significant reduction after 1 h CAP treatment compared with 0.5 h CAP treatment; ^iii^the number of bacteria on fabric surfaces with significant reduction after 0.5 h CAP treatment compared with control samples; ^iv^the number of bacteria on fabric surfaces with significant reduction after 1 h CAP treatment compared with 0.5 h CAP treatment; *the number of inactivated bacteria has a significant difference between different surfaces.

### 3.2 CAP inactivation of MDR bacteria on fabric surfaces

The four MDR strains on fabric surfaces were placed into the treatment device to assess the inactivation effect of CAP (Figure 1B; Table 2). After 30 min of CAP treatment, the survival of all MDR bacteria on fabric surfaces exhibited a notable decrease in comparison to the untreated samples. After 1 h of CAP treatment, no viable MDR bacteria were observed on the fabric surfaces. Furthermore, it is worth noting that the inactivation effect of CAP varied among MDR bacteria on different surfaces (Table 2). The inactivation effect of 30 min and 1 h of CAP treatment on CRAB and CRKP on fabric surfaces was more pronounced (*p* < 0.05) than that on culture dish surfaces. In the case of CRPA, after 30 min of CAP treatment, the inactivation effect was notably more conspicuous on culture dish surfaces than on fabric surfaces. These data indicated that CAP can inactivate MDR bacteria on fabric surfaces.

### 3.3 CAP inactivation of MDR bacteria in a simulation ward

A simulation ward was established to investigate the bactericidal effect of CAP technology in a real healthcare environment, and the active processes of CAP in the wards were tested (Supplementary Figure 5). As shown in Supplementary Figure 1, CAP affected MDR bacteria present on both culture dishes and fabric surfaces under the simulation ward settings. On the surface of culture dish at point A, the inactivation rates of CRAB, CRKP and CRPA peaked after 2 h of CAP treatment (95.4, 98.9 and 99.9%, respectively), while the inactivation rate of MRSA peaked after 8 h of CAP treatment (64.7%; Supplementary Figure 1A). On the surface of culture dish at point B, the inactivation rate of the four MDR bacteria by CAP reached a higher rate of inactivation (92.7, 98.2, 99.3 and 70.8%, respectively) after 2 h of treatment (Supplementary Figure 1B). On the surface of the fabric at point A, the maximum inactivation rates of CRKP and CRPA were reached at 2 h of CAP treatment (95 and 99.9%, respectively), CRAB peaked at 8 h of CAP treatment (98.3%), and the maximum inactivation rate of MRSA was reached at 24 h of treatment (96.7%; Supplementary Figure 1C). CRAB, CRKP and CRPA on the fabric surface at point B all reached more than 90% inactivation after 2 h of CAP treatment, whereas MRSA reached maximum inactivation (98.6%) only at 24 h of CAP treatment (Supplementary Figure 1D). These findings demonstrate that CAP exerts a sterilization effect on MDR bacteria on various surfaces in the simulation ward.

### 3.4 Comparison of sterilization effects between CAP and UV light

Given that UV light is generally acknowledged and widely used for hospital disinfection, UV light was installed at point A in the ward to compare the sterilization efficiencies of CAP technology and UV light. We use a movable UV disinfection vehicle, which has a power supply of 220 V ± 22 V, 50 Hz ± 1 Hz, an input power of 150 VA, and a wavelength of 253.7 nm. The key to ozone generation by UV light lies in the wavelength of UV, only when the wavelength of UV ≤ 200 nm (especially 185 nm), it can effectively decompose oxygen to generate ozone. The wavelength of the UV light we use is 253.7 nm, this wavelength can sterilize but not directly produce ozone. Regardless of whether the MDR bacteria covered culture dishes or fabric surfaces, the sterilization rate reached 99.00% after 2 h of UV exposure (Supplementary Figure 2). In the CAP treatment group, the sterilization rates of CRAB, CRKP, and CRPA on the surface of the culture dish reached 92.7, 98.2, and 99.3%, respectively, after 2 h of CAP treatment (Supplementary Figure 2A). On the fabric surfaces, the sterilization rates of CRKP, CRPA, and CRAB reached more than 90.0% after CAP treatment for 2 h, and the sterilization rate of MRSA was 87.1% (Supplementary Figure 2B). It can be seen that the efficacy of CAP in the sterilization of MDR bacteria was close to that of UV irradiation.

### 3.5 Effect of CAP on MDR bacteria surface morphology

**Figure 2:**
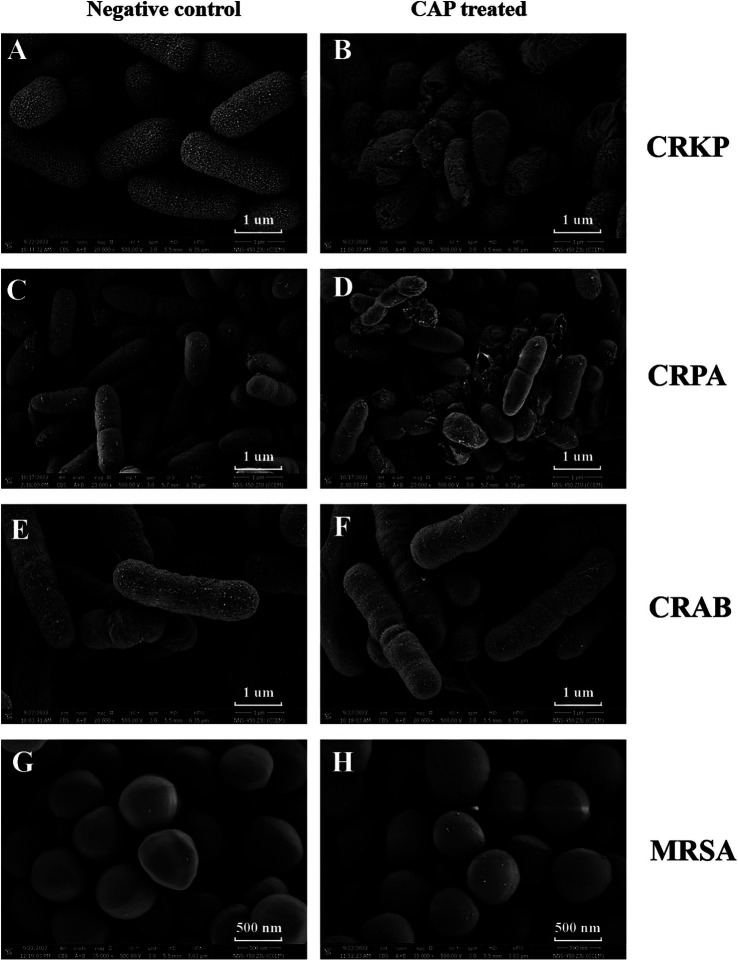
The CAP treatment affects the morphological characteristics of MDR bacterial pathogens. The CRKP **(B)**, CRPA **(D)**, CRAB **(F)**, MRSA **(H)** were exposed to CAP for 3 h. The untreated CRKP **(A)**, CRPA **(C)**, CRAB **(E)**, MRSA **(G)** served as a negative control. Then, the samples were harvested and observed under SEM. The magnification is indicated in each panel. CRKP, carbapenem-resistant *K. pneumoniae*; CRAB, carbapenem-resistant *A. baumanii*; CRPA, carbapenem-resistant *P. aeruginosa*; MRSA, methicillin-resistant *S. aureus*.

SEM was used to determine whether CAP treatment affected MDR bacterial surface morphology. SEM observations revealed that the surface of CRKP cells underwent changes after treatment with CAP. Upon observation, the surface structure of the CRKP control strain exhibited a uniform distribution with a villus-like pattern, and the bacterial cells appeared to be relatively dispersed (Figure 2A). After treatment with CAP, the surface structure of CRKP became blurred, the bacterial bodies ruptured, and the bacteria adhered to each other (Figure 2B). However, for CRAB, the surface structures of CAP-treated strains were similar to those of the untreated control strains (Figures 2C,D). In the case of the CRPA, certain differences were found between the experimental and control groups. The untreated CRPA strain exhibited structural integrity, with numerous surface secretions and a relatively dispersed arrangement (Figure 2E). CRPA-associated bacterial rupture and cell surface perforation were more frequently observed after CAP exposure (Figure 2F). In Gram-positive bacteria, untreated MRSA cells displayed a smooth surface with minimal secretions (Figure 2G). After CAP treatment, the surfaces of MRSA cells appeared rough (Figure 2H).

### 3.6 Effect of CAP on the internal structure of MDR bacteria

**Figure 3:**
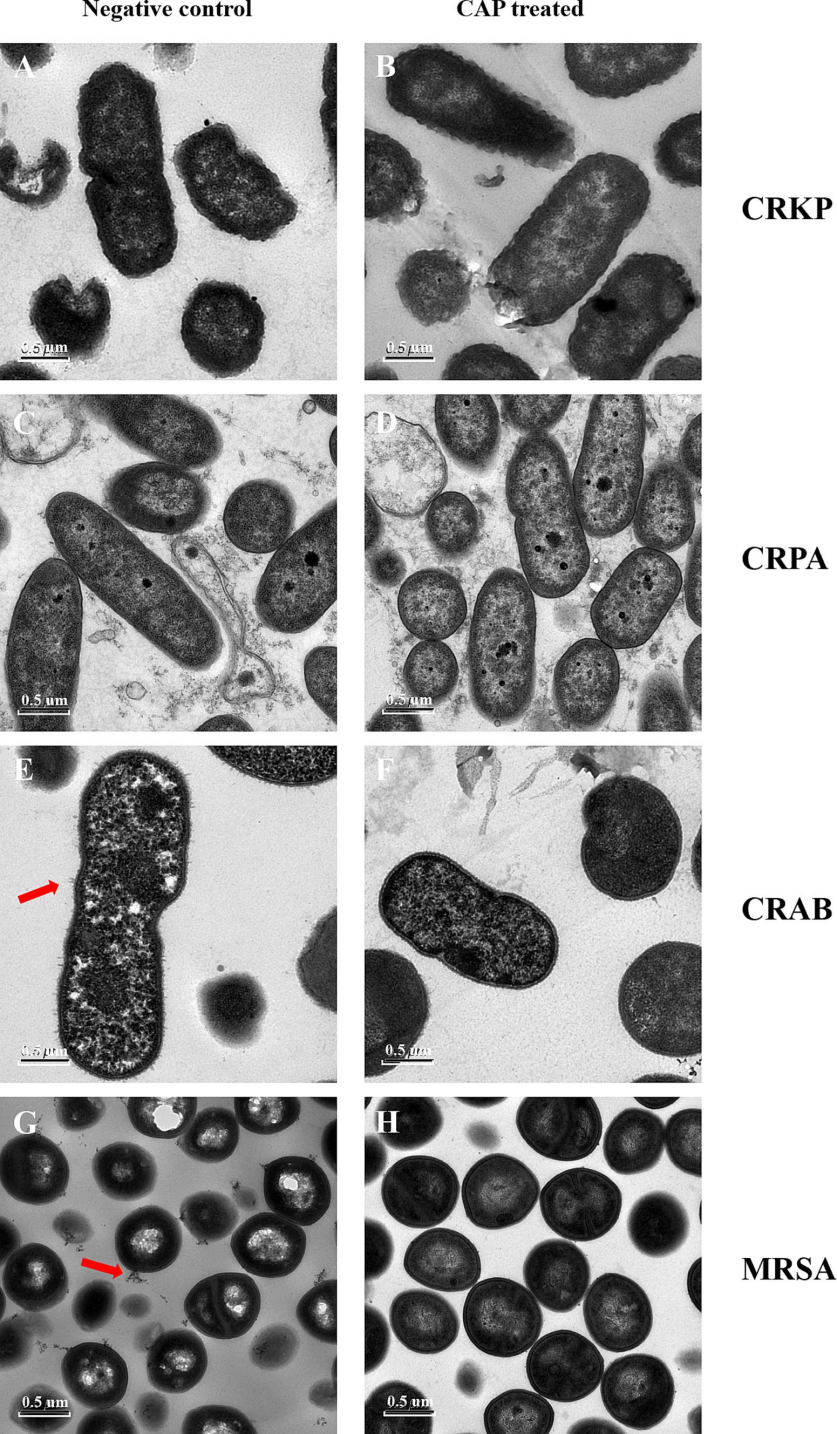
The CAP treatment affects the morphological characteristics of MDR bacterial pathogens. The CRKP **(B)**, CRPA **(D)**, CRAB **(F)**, MRSA **(H)** were exposed to CAP for 3 h. The untreated CRKP **(A)**, CRPA **(C)**, CRAB **(E)**, MRSA **(G)** served as a negative control. Then, the samples were harvested and observed under TEM. The magnification is 11,500X. CRKP, carbapenem-resistant *K. pneumoniae*; CRAB, carbapenem-resistant *A. baumanii*; CRPA, carbapenem-resistant *P. aeruginosa*; MRSA, methicillin-resistant *S. aureus*. The red arrows showed changes in fimbriae. The white arrows showed changes in exosomes.

TEM was performed to explore whether CAP treatment affected the internal structure of MDR bacteria. Subtle differences were observed between the MRSA and CRAB isolates. The CAP-treated samples showed a loss of fimbriae in CRAB and a reduction in exosomes in MRSA compared to the untreated groups (Figures 3C,D,G,H). For the CRKP and CRPA strains, no differences were observed in the cell membranes, walls, or other internal structures between the experimental and control groups (Figures 3A,B,E,F).

## 4 Discussion

Despite enhanced terminal cleaning, MDR bacteria can survive for months on the surface of the patient environment (19), partially because traditional cleaning methods are insufficient to control the spread of MDR bacteria. Our study demonstrated that CAP could inactivate the four most common MDR bacteria on nosocomial surfaces in hospital ward settings by destroying the pathogen structure. Furthermore, the effectiveness of CAP was contingent on treatment duration. The resistant phenotypes of the MDR bacteria investigated in our study differed from those reported previously. Nevertheless, our experimental findings demonstrate that CAP displays robust bactericidal activity against MDR bacteria, irrespective of their specific resistant phenotypes (20).

By expanding the experimental space, we found that CAP exerted an inactivated effect on MDR bacteria in the simulation ward. In this investigation, although the immediate sterilization efficacy of CAP within the simulation ward was slightly weaker than that of traditional UV methods over a brief period, the distinct advantage of CAP technology lies in its capacity to provide prolonged sterilization without impeding bed turnover in healthcare facilities. It was notable that as the incubation time extended to 24 h, there was a reduction in the bacterial count on the culture dish surfaces within the control group, compared with the initial bacterial culture. Thus, the sterilization rate exhibited a decline at 24 h relative to that observed at 8 h. Our study has some limitations. For instance, our experiments were conducted within a simulated ward environment, which, although designed to closely resemble a real ward, inherently differs from the actual clinical setting. As a next step, we recommend the initiation of a clinical trial for CAP sterilization in a real ward.

Although CAP plays a role in pathogen inactivation, its sterilization mechanism remains unclear. Many studies (21, 22) have revealed that the reactive species generated by plasma devices play an important role in the sterilization process. Depending on the plasma generation setup, humidity, pressure, and gas used, charged particles, atoms, highly ROS and reactive nitrogen can be generated, which can efficiently inactivate pathogens through direct permeabilization of the cell membrane or wall, DNA damage, and damage to intracellular proteins (23–25). The charged water particle-generating device used in this study mainly generated ROS, including •OH, ^1^O_2_, •O_2-_, and H_2_O_2_. Among these various ROS, ·OH stands out as the most potent oxidizing agent, yet it has a remarkably brief lifetime of only 10^−9^ s. Some researchers have reported that ·OH is an important factor in cytotoxicity (26, 27). This novel technology enhanced the generator to encapsulate the primary product, namely ·OH in water, allowing it to persist in the air for an extended duration compared to conventional negative ions. In this study, SEM and TEM were performed to explore the possible sterilization mechanisms associated with ROS. ROS disrupts the integrity of CRKP and CRPA cell membranes, potentially causing the leakage of intracellular components and microbial contraction. This was consistent with previous reports showing that ROS, especially ·OH and ^1^O_2_, could cause lipid peroxidation of the cell membrane, forming transient pores, followed by damage to cell membrane integrity (28). The bactericidal effects of ROS on MRSA and CRAB can be realized through the induction of DNA damage or the impairment of intracellular proteins. Previous reports have shown that when ROS accumulate in cells and exceeds a certain threshold, toxic ROS can be removed, and cellular antioxidant enzymes are also destroyed (29, 30). Moreover, a previous study indicated that CAP-treated *S. aureus* cells displayed a markedly reduced pigmentation phenotype (31), aligning with our experimental observations (although the specific data from our study are not presented here). The golden pigment in *S. aureus* plays a pivotal role in shielding cells from oxidative sterilization, indicating the influence of oxidative stress by ROS on MRSA (32, 33) Currently, the specific cellular targets of ROS remain unclear. Elucidating this intricate mechanism is crucial to developing an enhanced sterilization strategy that utilizes plasma for optimal applications.

In conclusion, the charged water particle-generating device used to generate CAP effectively inactivated MDR bacteria in both laboratory and simulated ward environments. Furthermore, the sterilization mechanism observed in this study suggests that ROS exert a bactericidal effect by disrupting the cell membrane integrity of CRKP and CRPA. Conversely, in the case of CRAB and MRSA, ROS predominantly aid sterilization by inducing damage to DNA or proteins. These data provide valuable insights into the sterilization mechanism of CAP against MDR bacteria and suggest a novel method for in-hospital disinfection.

## Data Availability

The raw data supporting the conclusions of this article will be made available by the authors, without undue reservation.
