# Supplementary material for: Inactivation of multidrug-resistant bacteria using cold atmospheric-pressure plasma technology
Source: Front Med (Lausanne). 2025 Mar 5;12:1522186. doi: 10.3389/fmed.2025.1522186 (PMC11920159; doi:10.3389/fmed.2025.1522186)
Supplement: Supplementary file 1 [file Supplementary_file_1.docx]

Supplementary Material


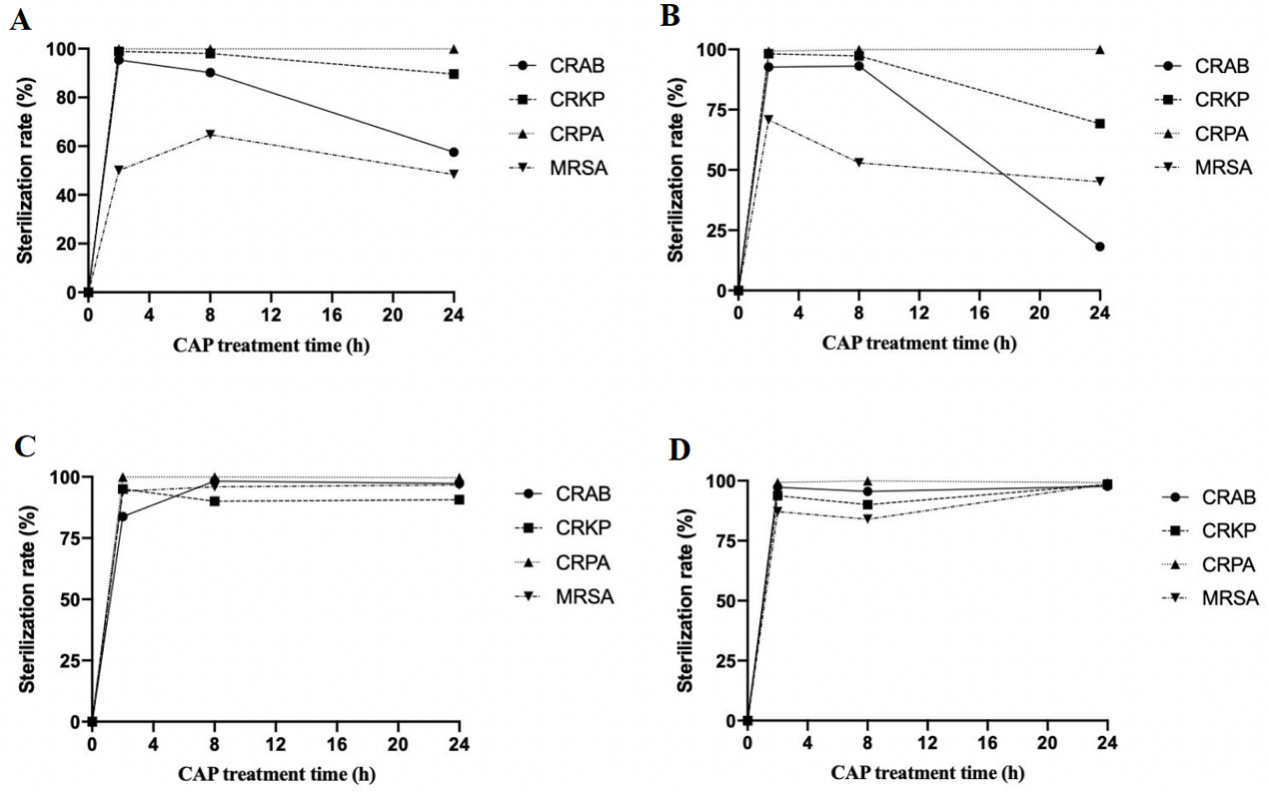


**Supplementary Figure 1.** Sterilization rate of CAP for four MDR bacterial species present on culture dish surfaces at point A (A), point B (B), and on fabric surfaces at point A (C), point B (D) after exposure to CAP identified by standard colony counting. CRKP, carbapenem-resistant *K. pneumoniae*; CRAB, carbapenem-resistant *A.baumanii*; CRPA, carbapenem-resistant *P. aeruginosa*; MRSA, methicillin-resistant *S. aureus*.


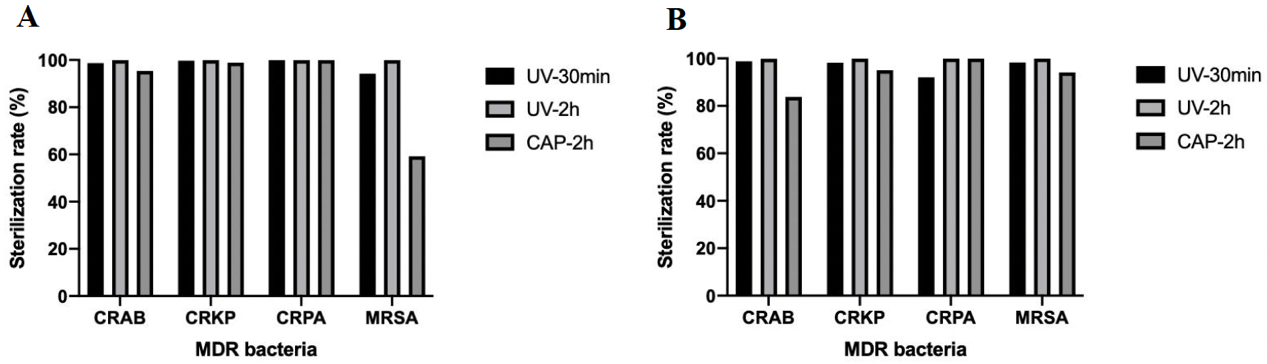


**Supplementary Figure 2.** Sterilization rate of UV and CAP for four MDR bacteria present on culture dish surfaces (A) and on fabric surfaces (B). CRKP, carbapenem-resistant *K. pneumoniae*; CRAB, carbapenem-resistant *A.baumanii*; CRPA, carbapenem-resistant *P. aeruginosa*; MRSA, Methicillin-resistant *S. aureus*.


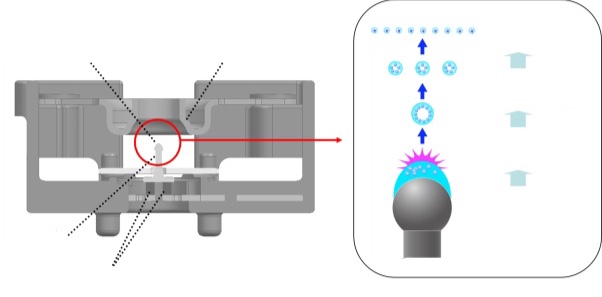


atomizing electrode

Condensate generation

opposite electrode

atomizing electrode

Peltier element

Condensate was produced at the front end of the electrode with the effect of Peltier element

converge atomized condensate at the tip

charged particle repeatedly split

nanometer water ions

**Supplementary Figure 3.** Structure of the charged water particle-generating device and generation principle of the device.


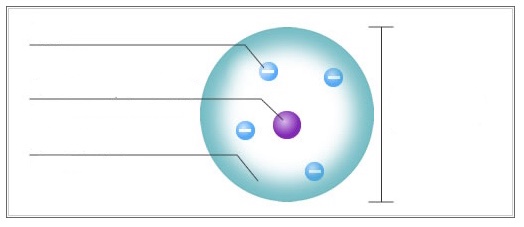


Electrons

5nm-20nm

Highly reactive component

Particles created from water

**Supplementary Figure 4.** Composition of nanometer water ions.


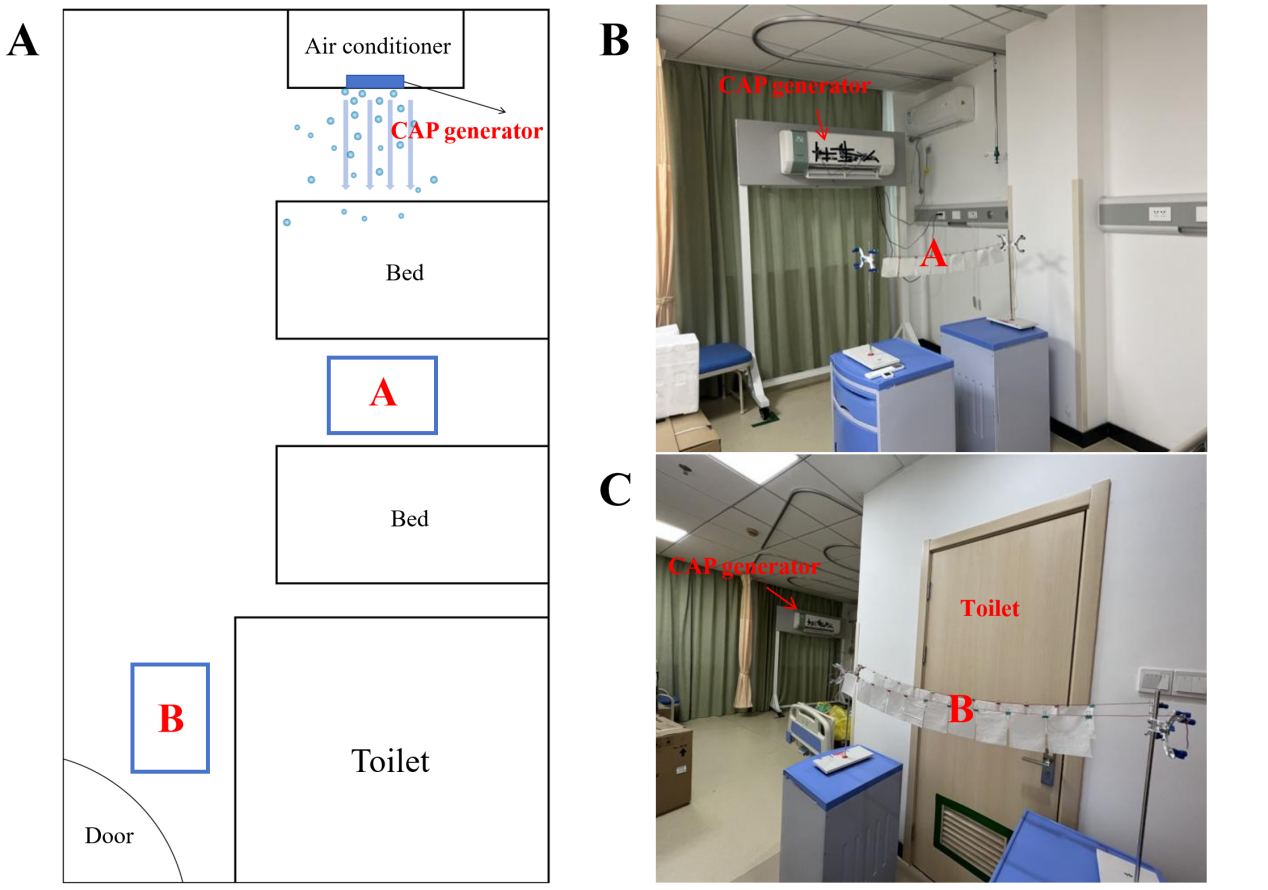


**Supplementary Figure 5.** Schematic diagram of simulated ward(A).Point A was positioned 1.5 meters away from the CAP device with a height set at 1.3 meters(B). Point B was located at the entrance of the ward with a height also set at 1.3 meters(C).
